# Addressing Stunting in Children Under Five: Insights and Opportunities from Nepal, Bangladesh, and Vietnam—A Review of Literature

**DOI:** 10.3390/children12050641

**Published:** 2025-05-16

**Authors:** Muhammad Yazid Jalaludin, Moretta Damayanti Fauzi, I Gusti Lanang Sidiartha, Collins John, Shamira Aviella, Edy Novery, Annisa Permatasari, Leilani Muhardi

**Affiliations:** 1 Department of Paediatrics, Faculty of Medicine, University Malaya, Kuala Lumpur 50603, Malaysia; 2 Child Health Department, Mohammad Hoesin Hospital, Palembang 30126, South Sumatra, Indonesia; 3 Child Health Department, University of Udayana, Denpasar 80361, Bali, Indonesia; 4 Department of Paediatrics, Jos University, Jos 930105, Plateau State, Nigeria; 5 Medical Affairs Department, Nestle Indonesia, Jakarta 12520, Indonesia; 6 Child Health Department, Bari Hospital, Palembang 30254, South Sumatra, Indonesia

**Keywords:** growth, child undernutrition, stunting, disparities interventions, outcomes, Bangladesh, Nepal, Vietnam

## Abstract

**Background**: Stunting remains a significant global health issue, particularly in low- and middle-income countries (LMICs). Globally, around 22% of children under five are affected, with high rates persisting in South and Southeast Asia. This review examines government-led programs in high-performing LMICs (Nepal, Bangladesh, and Vietnam) to identify key strategies and opportunities for effective intervention. **Methods**: A literature search was conducted on PubMed using keywords and Medical Subject Heading terms, including “stunting”, “child undernutrition”, “malnutrition” and the names of the three specified countries. Articles were evaluated for relevance based on their focus on stunting prevalence, risk factors, and interventions in these countries, without restrictions on publication date or language. **Results**: Stunting prevalence among children under five has significantly declined in Nepal, Bangladesh, and Vietnam over the past two decades, reflecting the impact of sustained nutrition and health interventions. Nepal reduced stunting from 55.8% in 2000 to 26.7% in 2022, Bangladesh from 54.7% to 26.4%, and Vietnam from 41.5% to 19.3%. Successful strategies included multisectoral approaches integrating nutrition-specific and nutrition-sensitive interventions, enhanced policy frameworks, and strong governance. Despite progress, challenges remain, such as high wasting prevalence in Nepal and disparities among marginalized communities in Vietnam, emphasizing the need for targeted, context-specific interventions. **Conclusions**: Effective stunting reduction requires multisectoral strategies addressing underlying, intermediate, and immediate determinants. Insights from Nepal, Bangladesh, and Vietnam highlight the importance of sustained government commitment, robust policies, and coordinated interventions. Adapting these successful strategies to local contexts can support stunting prevention and management, promoting healthier and more resilient communities.

## 1. Introduction

Growth is a dynamic process influenced by a combination of genetic and environmental factors (Table 1), leading to the progressive increase in both physical size and functional development of the body over time [1,2]. Genetic factors establish a child’s growth potential, influencing characteristics such as final adult height, body composition, and growth patterns. Stunting is often mistaken with short stature. The two most common types of short stature are Familial Short Stature (FSS) and Constitutional Delay of Growth and Puberty (CDGP) [3,4]. A child with FSS is diagnosed when he or she is growing at a normal rate (following his or her curve) and one or both parents are short—that is, the mother is 5′1″ (~155 cm) or shorter and/or the father is 5′5″ (~165 cm) or shorter. Standard height in some populations are as follows: Nepal: male: 5′3.5″ (161.7 cm) and female: 4′11″ (150.4 cm); Bangladesh: male: 5′4″ (162.1 cm) and female: 4′11″ (150.3 cm), Indonesian men: the normal height is typically cited as around 5′5.6″ (166.2 cm) and for women around 5′0.8″ (154.3 cm), Vietnamese male: 5′6″ (168.1 cm) and women: 5′1.5″ (156.2 cm); and the Netherlands: male: 6′0.5″ (183.8 cm) and female: 5′7″ (170.7 cm) [5].

Overall, in contrast, children with CDGP are diagnosed when the child is usually healthy and growing normally but slightly below the curve. However, neither parent is short, and in most cases, one parent was a late maturer [3,4]. Environmental factors, including adequate nutrition, socioeconomic conditions, healthcare access, and psychosocial stimuli during critical growth periods, also play crucial roles in achieving growth potential [1,2].

Normal growth in children is a key indicator of their overall health and nutritional status [20]. It is assessed using measurable parameters such as height, weight, and head circumference, alongside developmental milestones, all of which follow predictable age- and sex-specific patterns [21]. Chronic malnutrition, particularly during the first 1000 days of life, can result in faltering growth, wasting and stunting, negatively affecting physical and cognitive development [22,23,24]. Stunting, in particular, is primarily driven by inadequate nutrition but is also exacerbated by recurrent infections and socioeconomic challenges [20,21,22].

The World Health Organization (WHO) defines stunting as a height-for-age Z-score (HAZ) of more than two standard deviations (SD) below the median of the WHO growth standards [25]. This standardized definition facilitates the consistent comparison of stunting data across countries and time periods. Regular monitoring and plotting of HAZ on the WHO growth chart enables early detection and management of children at risk of growth abnormalities [21,26,27]. Nonetheless, delayed diagnosis and treatment of growth faltering remain common, limiting the recovery potential of at-risk children. Healthcare workers play a pivotal role in ensuring consistent monitoring, providing nutritional support, and implementing timely corrective measures to promote optimal growth and development [26,27,28].

**Figure 1:**
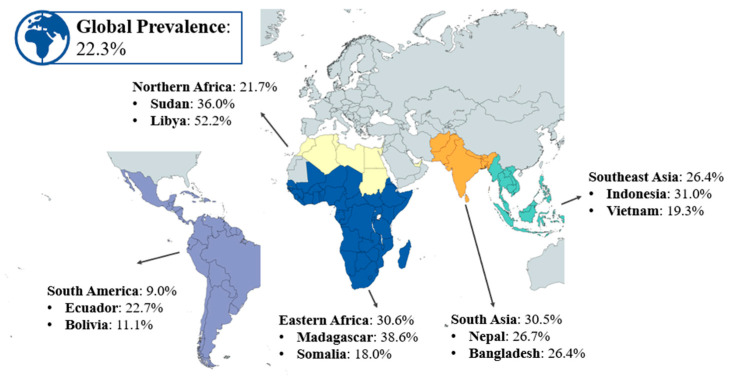
Prevalence of stunting across various regions in the world in 2022. Figure illustrates the stunting rates among children aged below five years across various regions, including South America, Northern Africa, Eastern Africa, South Asia, and Southeast Asia [26].

While significant progress has been made globally to reduce stunting prevalence, it remains a pressing issue in low- and middle-income countries (LMICs) [20]. In 2022, 22.3% of children under five years of age were stunted globally (Figure 1) [29]. While this represents a significant decline from 33% in 2000, stunting persists as a major challenge in certain regions, particularly in Eastern Africa, South Asia, and parts of Southeast Asia [29]. The progress has been hindered by factors such as inadequate maternal nutrition, poor infant feeding practices, persistent poverty, food insecurity, political instability and limited access to healthcare [30]. According to the 2023 Joint Child Malnutrition Estimates (JME), only about one-third of countries are likely to achieve the 2030 target of halving the number of stunted children [29]. Therefore, greater focus and targeted interventions will be needed to meet the global goal of reducing stunting to 14% by 2030 [29].

This review examines learnings from stunting reduction programs in high performing countries achieving ≥30% reductions in stunting over recent decades such as Nepal, Bangladesh and Vietnam. These insights can guide other countries in adapting successful strategies to local contexts, promoting improvements in child growth and development.

## 2. Methods

A literature search was conducted to identify relevant studies and reports on stunting prevalence and government programs targeting stunting in children under five in Nepal, Bangladesh, and Vietnam. PubMed was used as the primary platform for retrieving peer-reviewed articles and reports, and several sources of grey literature were also searched. The search strategy combined keywords and Medical Subject Headings (MeSH) terms, including ‘child undernutrition’, ‘intervention’, ‘malnutrition’, ‘stunting’, ‘Bangladesh’, ‘Nepal’, and ‘Vietnam’.

The relevance of each article was critically evaluated in the context of the objectives of this review—articles addressing the prevalence of stunting, its associated risk factors, and the design, implementation, and outcomes of stunting intervention programs in the specified countries. Inclusion criteria included studies focusing on stunting in children under five years; those discussing stunting-related opportunities and challenges in Nepal, Bangladesh, and Vietnam; and studies providing quantitative or qualitative data on interventions, determinants, or outcomes of stunting. Exclusion criteria included studies on non-relevant countries or regions; and those addressing stunting in individuals over five years of age. No restrictions were applied concerning the date of publication or language to ensure a broad and inclusive scope of available literature. Additionally, cross-referencing from the retrieved articles was manually reviewed.

## 3. Results and Discussion

### 3.1. Search Results and Included Articles

The literature search yielded a total of 5449 review articles, reports, and scientific papers. However, only 55 studies were included in the final analysis which represented diverse sources, including guidelines, systematic and non-systematic reviews, randomized controlled trials, observational studies, and reports from governmental and organizational programs.

The study limitation: The results presented in this paper are not a systematic review which may have resulted in some missing articles or other references. In addition, there is only one database being used which could result in many relevant articles not being retrieved. Thus, the suggestions based on the retrieved insights should be adjusted to the local context in each country.

### 3.2. Determinants of Stunting

**Figure 2:**
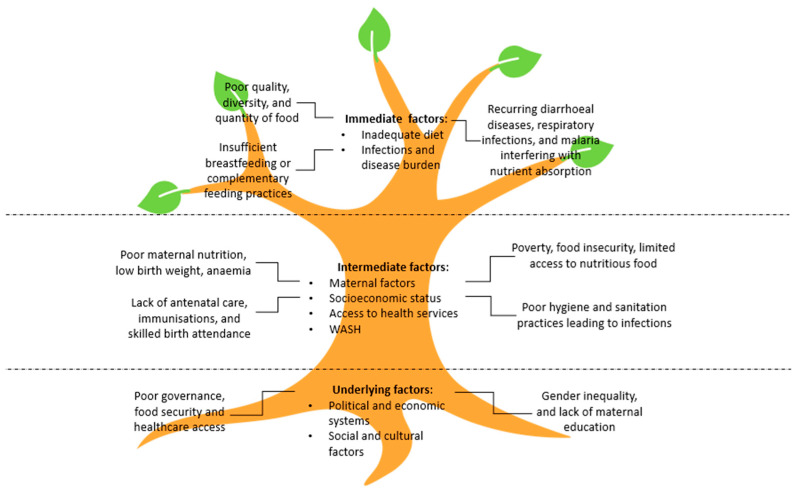
Determinants of stunting. This figure conceptualized stunting as a tree, where the roots represent the underlying factors, the trunk symbolizes the intermediate factors, and the leaves depict the immediate factors contributing to the condition. WASH, water, sanitation and hygiene [31].

Stunting results from the combination of underlying, intermediate, and immediate factors that disrupt growth, which may manifest as stunting (Figure 2) [31].

### 3.3. Underlying Factors

Underlying factors are the structural and systemic conditions that shape the intermediate and immediate factors, and can be categorized into governance, resources, and cultural norms. Political and economic systems influence national policies on food security, healthcare, and social protection, which directly impact stunting rates. Cultural norms, such as gender inequality and harmful child-feeding practices, further exacerbate the problem [32]. For example, in some cultures, giving newborns pre-lacteal food and taboos surrounding early initiation of exclusive breastfeeding lead to inappropriate young child feeding practices that can contribute to stunting [33,34].

### 3.4. Intermediate Factors

Intermediate factors indirectly influence child health and nutrition. Poor maternal nutrition during pregnancy and anemia lead to low birth weight, which is a significant risk for stunting in infancy and early childhood. Socioeconomic constraints limit access to nutritious foods, clean water, and healthcare services, forcing households into food insecurity and poor health outcomes. Limited access to healthcare, particularly antenatal and postnatal services, worsens maternal and child health. Inadequate water, sanitation, and hygiene (WASH) can exacerbate immediate factors, such as infectious diseases, which impair nutrient absorption and contribute to faltering growth and wasting [31].

### 3.5. Immediate Factors

Immediate causes of stunting are directly related to a child’s nutrition and health. Inadequate intake of animal protein, particularly milk during the critical first 1000 days, deprives children of essential amino acids and bioactive factors needed for growth [35,36]. Poor breastfeeding practices, such as insufficient exclusive breastfeeding, further exacerbate nutritional deficits. In many low-resource settings, complementary feeding practices, i.e., when solid foods are introduced, are often poor in micronutrients and essential fats, further contributing to stunting. Additionally, infectious diseases, including diarrhea, respiratory infections, and malaria, hinder nutrient absorption, increase energy demands, and worsen malnutrition. These immediate factors often initiate faltering growth [37].

### 3.6. Children at Risk of Stunting

#### 3.6.1. Faltering Growth

Faltering growth occurs when a child fails to gain weight at an expected rate. It is often an early sign of poor nutrition and/or other underlying health issues and can be a precursor to stunting if not addressed. Faltering growth is especially of concern in infants and young children as it can impair growth and development, and recurrent episodes of faltering growth led to stunting [38]. From the Young Lives cohort, children who experienced faltering growth (not stunted at age one year but stunted at age eight years) tended to perform worse in cognitive achievement than children who were never stunted [24,39], emphasizing the need for early life growth monitoring. The current WHO definition of faltering growth provides a pragmatic approach to recognizing faltering growth in children but lacks a timeframe for the period of faltering growth that excludes acute weight loss typical of vomiting/diarrhea and neonatal weight loss [38]. An expert group has proposed defining faltering growth as a fall in the weight-for-age z score of ≥1.0 that occurs over a period of ≥one month and does not include the first two weeks after birth [38].

#### 3.6.2. Wasting

Wasting or low weight-for-height is a form of acute malnutrition and reflects recent weight loss or failure to gain weight due to insufficient food intake, infections, or other health issues. Unlike stunting, which is a long-term process, wasting is typically a more immediate response to nutrition and health crises. Wasting and stunting often occur together, but wasting can also be a temporary condition that might not result in long-term stunting if corrected quickly. However, repeated episodes of wasting increase the likelihood of stunting due to prolonged or repeated nutritional and health stresses [23,40,41,42]. As with stunting, wasting in the first 1000 days of life is linked to delays in fine motor development (bringing objects to the mouth, eating with hands, and palmar grasp) and cognitive milestones (smiling, following objects with the eyes, reacting to sound stimuli, and social interaction) [23].

### 3.7. Dynamics of Stunted Growth in Children

**Figure 3:**
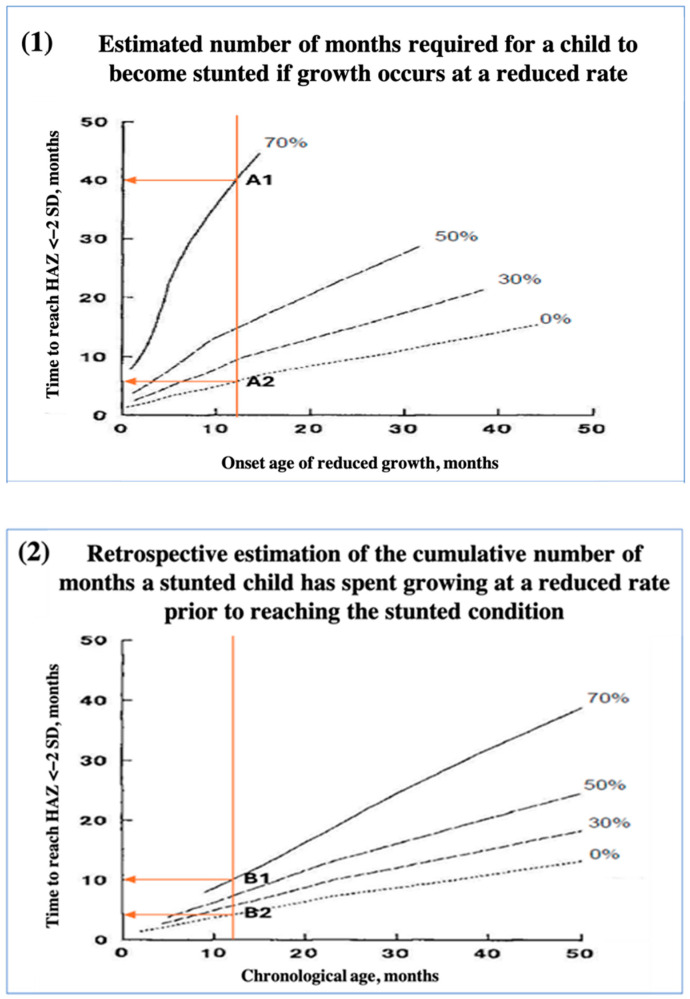
Dynamics of stunted growth in children. Graphs illustrate the time necessary for a child of a certain age to become stunted if growth occurs at a reduced rate (0%, 30%, 50%, and 70% of the standard rate).

Stunted growth in children signifies a failure to achieve age-appropriate linear growth. Catch-up growth requires an accelerated growth rate beyond that of peers. If a normal growing child of a certain age reduces their growth rate, the duration required to be classified as stunted increases with age. For instance, if growth ceases at 12 months, stunting occurs after nearly 6 months (at 17.7 months of age), whereas cessation at 36 months takes 13 months (at 49 months of age) to reach the same threshold (Figure 3) [36]. As children grow older and fall further behind, the time required to achieve catch-up growth increases, and complete recovery may become unattainable owing to limited growth potential. Prolonged and accelerated growth necessitates long-term improvements in the child’s environment, including consistent access to adequate nutrition and healthcare. Short-term interventions are insufficient to ensure sustained catch-up growth and may result only in transient improvements. This underscores the importance of early and sustained efforts to address the factors contributing to stunting [43].

Based on Figure 3(1), the estimated number of months required for a child to become stunted if growth occurs at a reduced rate, and Figure 3(2), the retrospective estimation of the cumulative number of months a stunted child has spent growing at a reduced rate prior to reaching the stunted condition. This would means if a child grows at 70% of the standard rate starting at age 12 months, they may become stunted after 40 months, reaching stunting at age 52 months. In contrast, if the child ceases growth entirely at age 12 months, they may become stunted within 6 months, at 18 months of age. For a child diagnosed as stunted at 12 months, they would have spent 10 months growing at 70% of the standard rate to reach a HAZ < −2 SD. Alternatively, the same child would have spent 5 months with no growth to be classified as stunted by 12 months. HAZ, Height-for-Age Z-score; SD, standard deviation [43].

### 3.8. Window of Opportunity

While catch-up growth can mitigate some of the negative consequences of stunting, its effectiveness largely depends on the timing of intervention. The first 1000 days of life represent a critical period for both physical and cognitive development. While weight recovery is often achievable, height recovery is typically less complete, leaving stunted children shorter than their peers despite reaching normal weight levels [39].

A longitudinal cohort study involving 8062 children from Ethiopia, India, Peru, and Vietnam demonstrated that addressing stunting in early childhood can improve cognitive and educational outcomes. Growth improvements beyond early childhood were associated with better schooling outcomes and higher cognitive achievement at eight years of age, compared with persistently stunted children [24]. Complementary evidence on Indonesian children’s cognitive function suggests that the absence of catch-up growth following poor early growth poses notable risks to cognitive development [44].

While the first 1000 days of life represent a critical window for intervention, targeted nutrition and health programs for pre-primary and early primary school-aged children can further mitigate the long-term effects of stunting. This evidence highlights the need for sustained, age-appropriate interventions to optimize both physical growth and cognitive development in vulnerable populations [24].

### 3.9. Consequences of Stunting on Cognitive Function and Human Capital

Stunting affects not only physical growth but also has profound and long-term consequences for cognitive function, intelligence, and educational outcomes. Stunting during the first 1000 days of life is associated with reduced cognitive development, including lower IQ scores [45,46]. Research suggests that severe stunting in the first 1000 days of life can reduce IQ scores by approximately 11 points by the time these children reach eight years of age compared to children who were never stunted [39].

**Figure 4:**
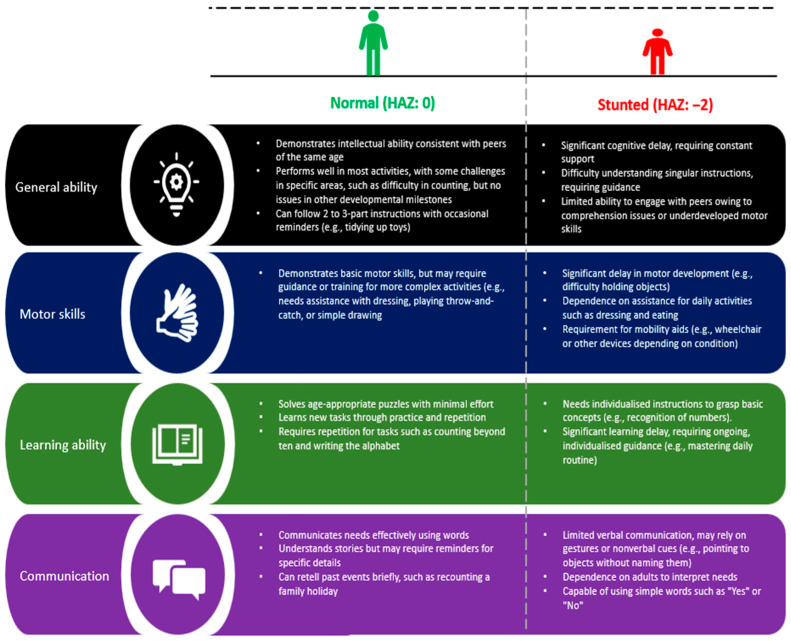
Comparison of various cognitive abilities between normal and stunted children aged 5 years. A comparison of the mid- to long-term consequences of stunting on general ability, motor skills, learning ability, and communication in children aged five years with normal growth versus those who are stunted [39].

Stunted children are more likely to experience delays in key cognitive domains, such as language development, memory, learning capacity, and concentration, all of which contribute to poorer academic performance [45,47]. Beyond academic challenges, stunted children face a higher likelihood of social interaction difficulties, emotional challenges, and behavioral problems. These cognitive and behavioral impairments often persist into adulthood, limiting social mobility, economic opportunities, and overall quality of life [17]. Stunting can also affect motor skills and communication, significantly affecting long-term learning potential and adaptability (Figure 4) [39].

Childhood stunting has long-term implications on human capital, including impaired physical growth, lower educational attainment, reduced workforce productivity, and decreased wages. In LMICs, childhood stunting costs private sector losses of at least USD 135.4 billion annually, equivalent to 0.01–1.2% of the gross domestic product, with the manufacturing, garment, and food sectors being the most affected [48]. Conversely, every unit increase in HAZ in the first 1000 days of life is associated with a 0.22 SD improvement in cognitive function among children aged 5–11 years [43], an additional 0.78 years of schooling [47], and higher reading (0.28 SD) and nonverbal cognitive test scores (0.25 SD) [47]. Investments in nutrition-specific interventions in LMICs yielded substantial economic benefits, with benefit–cost ratios exceeding one and returns ranging from 100% to 8100% for every dollar invested [48].

## 4. Insights from High-Performing LMICs

### 4.1. Stunting Prevalence and Progress in LMICs

In LMICs such as Nepal, Bangladesh, and Vietnam, the prevalence of stunting among children under five years of age has historically been high, with rates exceeding 50% in some regions three decades ago [49]. However, over the past 22 years, Nepal had reduced stunting prevalence from approximately 55.8% in 2000 [25] to 26.7% in 2022 [29]; in Bangladesh, it has declined from 54.7% to 26.4%, and in Vietnam from 41.5% to 19.3% [25].

Bangladesh also has made some progress towards the wasting reduction target. However, 9.8% of children aged < 5 years are still affected, which remains higher than the Asia regional average of 8.9% [50]. Vietnam has also made progress, achieving a wasting prevalence of 5.2%, which is lower than the regional average [51]. Recognizing this, these countries have implemented many nutrition-based programs aimed at addressing the three determinants of stunting.

### 4.2. Multisystem Approach to Combat Stunting

**Figure 5:**
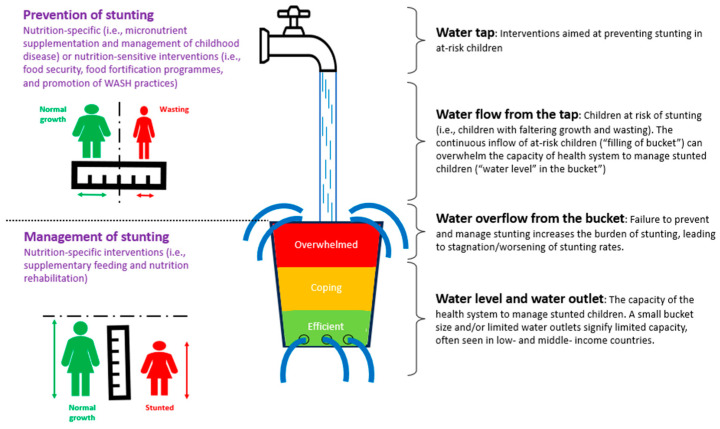
A multisystem approach to stunting prevention and management.

This review identified that a multisystem approach comprising nutrition-specific and nutrition-sensitive programs is needed to address stunting and its complex causes (Figure 5). While existing interventions have proven effective, the continuous influx of at-risk children may place strain on the health system, necessitating upstream measures to prevent faltering growth [37].

The water tap and bucket analogy depicts the complexities of stunting and the need for integrated strategies targeting both prevention and management. The at-risk population represents children experiencing faltering growth, characterized by slower-than-expected weight gain for age and sex. Prevention of stunting includes nutrition-specific interventions and nutrition-sensitive interventions. Management of stunting primarily involves nutrition-specific interventions. Overflowing of the bucket signifies the health system’s inability to manage the burden, emphasizing the need for upstream interventions [37].

Nutrition-specific programs directly address the immediate causes of malnutrition, including undernutrition and micronutrient deficiencies, targeting both prevention and management. Key interventions include micronutrient supplementation (e.g., iron, folic acid, and vitamin A), promotion of optimal breastfeeding, complementary feeding practices, nutrition intervention, supplementary feeding, and nutrition rehabilitation [6,20,52].

On the other hand, nutrition-sensitive programs indirectly address prevention by tackling the intermediate and underlying causes of malnutrition. These programs integrate nutrition goals into broader development sectors, including agriculture, education, social protection, and WASH. By enhancing food security, improving access to clean water and sanitation, promoting education, and providing social safety nets, these initiatives create an enabling environment for addressing stunting [53].

### 4.3. Country-Specific Approaches to Stunting Reduction

Nepal has implemented various stunting reduction programs targeting children aged < 5 years, strengthening multisectoral coordination, policy frameworks, and government capacity to deliver nutrition services at both central and local levels. Community-based interventions have increased early breastfeeding initiation, expanded vitamin A supplementation, and improved dietary diversity among young children. Additionally, nutrition-sensitive strategies, such as livelihood support and agricultural interventions, have contributed to poverty reduction and household food security [25,54,55,56,57,58]. However, wasting prevalence among children aged < 5 years remains high, possibly due to challenges such as shortages of skilled human resources. This includes limited capacity among healthcare workers to deliver effective nutrition counseling and supervision at health facilities, as well as gaps in agricultural extension services that hinder the implementation of nutrition-sensitive interventions [59,60].

Bangladesh has implemented a range of programs addressing both nutrition-specific and nutrition-sensitive drivers to combat malnutrition and stunting. These initiatives have integrated essential interventions such as micronutrient supplementation, maternal and child nutrition services, food fortification, and school-based nutrition programs, alongside broader efforts targeting food security, WASH practices, and BCC Behavioral Change Communication; WASH, Water, Sanitation and Hygiene (BCC) [50,61,62,63]. Although the multisectoral approach has contributed to improved nutrition outcomes, there were challenges such as limited coordination among stakeholders, gaps in implementation, and resource constraints [64].

Vietnam has incorporated nutrition-specific and nutrition-sensitive strategies to address malnutrition and stunting, including micronutrient supplementation, school milk programs, and poverty reduction measures, to enhance food security and healthcare access. Additionally, mass media campaigns and community health workers have driven improvements in promoting female education and healthcare access [65,66,67]. However, challenges persist, particularly among marginalized ethnic communities where stunting rates remain stagnant, highlighting disparities in nutrition outcomes. Vietnam’s experience underscores the importance of targeted, inclusive nutrition strategies to address both broad and community-specific needs [68].

Table 2 summarizes the primary interventions assessed, while Table 3A–C provides an overview of the key government-led stunting programs and their outcomes.

## 5. Approaches to Managing Stunted Children

Although the government programs primarily focused on stunting prevention, several interventions may also help mitigate the effects of stunting in already affected children by promoting catch-up growth and addressing nutritional deficits. One key strategy is supplementary feeding; A quasi-experimental study in Indonesia found that supplementary feeding significantly increased both height and weight in stunted toddlers, indicating its effectiveness in promoting growth recovery [80]. Similarly, a systematic review evaluating supplementary feeding interventions in disadvantaged children aged three months to five years reported improvements in health and well-being, particularly among those who were poorer and less well nourished [81].

Dairy may be beneficial due to its high digestibility-corrected amino acid score, which helps close amino acid gaps common in monotonous diets as seen in Africa and Asia, and poorer populations that are more susceptible to infections [82]. It uniquely stimulates plasma insulin-like growth factor 1, promoting amino acid uptake and growth. Additionally, dairy is calorie-dense and rich in essential micronutrients and calcium, which support bone length and strength [35,83]. Its nutrient density, taste, and familiar texture make it well suited for young children with small stomachs who struggle to consume large quantities of nutrient-sparse foods [83]. Dairy consumption has been lined to faster growth in early childhood [84,85].

In addition to supplementary feeding, nutrition rehabilitation has also demonstrated positive outcomes for stunted children. A study conducted in eastern Nepal followed children discharged from a nutritional rehabilitation center for one year, revealing significant catch-up growth with greater weight and height gains compared to control groups [86].

## 6. Suggested Interventions Addressing the Determinants of Stunting

Through the analysis of successful stunting reduction efforts in the three high-performing countries, this review identified potential program strategies applicable to countries with similar challenges.

### 6.1. Underlying Determinants (Socioeconomic Factors)

Sustained stunting reduction requires strengthened multisectoral coordination and governance through the expansion of national nutrition policies. Effective intervention depends not only on well-designed initiatives but also on documented policies and strong government commitment to ensure scalability and sustainability. All three countries have demonstrated this commitment through decades of continuous national programs. For instance, Nepal’s Multisector Nutrition Plan (2013–2022) and Bangladesh’s National Plan of Action for Nutrition (2016–2025) reflect long-term investment in nutrition governance. Similarly, Vietnam’s National Nutrition Strategy (2011–2020) addresses the triple burden of malnutrition (undernutrition, overnutrition, micronutrient deficiencies) through robust governance and multisectoral coordination [30,61,87,88,89,90]. The Scaling Up Nutrition (SUN) movement, a global initiative launched in 2010 to combat malnutrition, highlights that governance buy-in, supported by strong policies and adequate resource allocation, is crucial for impactful interventions [87,91]. Policies should focus on poverty alleviation, food fortification, and equitable access to healthcare and education [89]. Sustained advocacy efforts involving communities, schools, and policymakers are key to maintaining political commitment and resource allocation over the long term.

### 6.2. Intermediate Determinants (Health, Environment, and Community Factors)

Enhancing sanitation infrastructure to eliminate open defecation, a prevalent issue in many LMICs, is critical for reducing stunting by minimizing exposure to enteric infections and improving overall child health. Expanding and training healthcare volunteers can strengthen maternal and child health services, ensuring greater access to prenatal care, immunization, and nutrition support. Additionally, scaling up school feeding and milk supplementation programs, combined with integrating nutrition education into the curriculum, can promote adequate childhood nutrition while fostering lifelong healthy eating habits, ultimately contributing to sustained reductions in malnutrition and stunting [49,92].

### 6.3. Immediate Determinants (Nutrition and Health Factors)

Micronutrient supplementation and supplementary feeding for pregnant women and children, are essential for addressing nutrient deficiencies that impact fetal and early childhood growth. Strengthening exclusive breastfeeding promotion through BCC strategies can enhance breastfeeding practices, improving infant nutrition and immune protection. Additionally, management of childhood illnesses is crucial for reducing the burden of diarrheal diseases and infections, which significantly contribute to faltering growth. These interventions collectively support optimal early childhood development and help mitigate the immediate determinants of stunting.

## 7. Conclusions

Addressing stunting in children aged < 5 years requires comprehensive, multisectoral strategies that target the underlying, intermediate, and immediate determinants of growth failure. This review highlights insights from Nepal, Bangladesh, and Vietnam, where sustained government commitment, robust policy frameworks, and coordinated interventions have significantly reduced stunting.

Governance buy-in and long-term policy support are crucial for sustainable progress, with a focus on food fortification, poverty alleviation, healthcare access, and education. Improving sanitation infrastructure and promoting hygiene practices are essential for minimizing exposure to enteric infections, which are key intermediate determinants of stunting.

Nutrition-specific interventions, such as micronutrient supplementation and breastfeeding promotion, address immediate determinants by directly enhancing maternal and child nutrition. These interventions are particularly vital during the first critical 1000 days of life, when growth and cognitive development are most sensitive to nutritional and environmental influences.

Lessons from the above-mentioned countries underscore the importance of coordinated community-based approaches that link nutrition goals with broader social and healthcare strategies. Adapting these successful models to local contexts can support both prevention and management of stunting, ultimately fostering healthier and more resilient populations.

## Figures and Tables

**Table 1:** Influencing factors on child growth.

Factors	Impact on Child Growth
Nutrition and environmental influences	A balanced diet rich in macronutrients (i.e., proteins, fats, carbohydrates) and micronutrients (i.e., vitamins, and minerals), is essential in supporting cell division, tissue repair, and overall growth and development [6] Environmental conditions, including exposure to toxins, pollutants, and stressors (i.e., nutritional deficiencies, infectious diseases), can negatively impact growth [7] Maternal nutritional status directly influences fetal development, birth outcomes, and the child’s nutritional reserves during early life [8]
Genetic disorders	Atypical growth factors can occur in certain genetic conditions, such as Noonan, Turner or Prader–Willi syndromes [9]
Hormonal regulation	Growth and thyroid hormones are vital for stimulating tissue growth and regulating metabolic processes, with growth hormone secretion peaking during childhood and thyroid hormone deficiency impairing growth and development [10,11] Puberty typically begins at a skeletal age of approximately 11 years in girls and 13 years in boys, causing changes in bone density, muscle mass, and body fat composition through the actions of sex and growth hormones. During the pubertal growth spurt, girls attain peak height velocity of 9 cm/year at age 12, while boys reach 10.3 cm/year at age 14 [12]. Children born small-for-gestational-age experience an earlier onset of puberty (1.6–2.3 months for girls; 0.6 months for boys) than peers with normal birth weight [13]
Psychosocial factors	Family environment, emotional support, and socioeconomic status may impact a child’s growth and development, with supportive environments promoting better outcomes and neglect or emotional stress potentially leading to growth delays and psychological challenges [14,15]
Physical activity, sleep, and overall health	Regular physical activity and adequate sleep are essential for healthy growth, while the absence of acute and chronic infections is crucial, as the metabolic demands of infections divert resources needed for growth [16,17,18]. Furthermore, there is a bidirectional relationship between infection and stunting, where malnutrition predisposes to infection, and infection, in turn, exacerbates malnutrition, creating a cyclical interaction that hinders development [19]

**Table 2:** Key interventions in government stunting programs in Nepal, Bangladesh, and Vietnam among children aged < 5 years.

	Nepal	Bangladesh	Vietnam
**Underlying determinants**			
**Food security and food fortification programs, BCC initiatives, multisectoral collaboration**	✓ [69,70,71,72]	✓ [55,61,62,73]	✓ [66,67,74]
**Intermediate determinants**			
**Community-based nutrition programs, promotion of WASH practices, healthcare worker training**	✓ [69,70,72,75]	✓ [61,62,73]	✓ [66,68,74,76]
**Immediate determinants**			
Micronutrient supplementation *, exclusive breastfeeding, maternal and child nutrition services ^†^	✓ [69,70,72]	✓ [50,61,62,63]	✓ [74,77]
**Supplementary feeding (i.e., milk supplementation)**		✓ [78]	✓ [66]
**Management of childhood diseases**	✓ [55,60,70]	✓ [79]	

* i.e., providing vitamin A, iron-folate, iodine, zinc to children and pregnant women. ^†^ promoting appropriate infant and young child feeding practices. Behavioral Change Communication (BCC); Water, Sanitation and Hygiene (WASH).

**Table 3:** (**A**): Programs implemented by the Nepal government to address stunting; (**B**): Programs implemented by the Bangladesh government to address stunting; (**C**): Programs implemented by the Vietnam government to address stunting.

(**A**)		
**Programs**	**Key interventions**	**Key outcome measures**
**MSNP and MSNP-II (2013–2022)** [51] To reduce stunting in children aged < 5 years to below 29% and wasting to below 5%. To enhance maternal and child nutrition during the first 1000 days of life To strengthen coordination and governance	*Nutrition-specific [30]:* IYCF practices Micronutrient supplementation Management of acute malnutrition through nutrition rehabilitation centers *Nutrition-sensitive [30]:* Agriculture and food security initiatives WASH programmes Education and social protection measures	Achieved its stunting reduction target, lowering prevalence to 26.7%; however, 12.0% of children under five remain affected by wasting [25,30,57] Improved management and delivery of nutrition programs [55] Improved policies, strategic plans, and strengthened multisectoral coordination at national and local levels [55].
**FCHV Programme (launched in 1988)** To extend healthcare services by engaging local women to deliver nutrition education and interventions in remote communities [69]	*Nutrition-specific [55]:* Micronutrient supplementation IYCF promotion Management of acute malnutrition Maternal and child nutrition education *Nutrition-sensitive [55]:* WASH activities Community mobilization and advocacy	Achieved 95% biannual vitamin A supplementation coverage for children aged 6–59 months [69] Increased early initiation of breastfeeding (within the first hour) from 18% in 1996 to 55% in 2016 [69] Distributed over 818,000 packets of oral rehydration solution to treat diarrheal cases in children aged < 5 years [69] Reached 84% deworming coverage by 2006 [69]
**Suaahara Project (2011–2016)** [72] To improve maternal, infant, and young child nutrition To enhance household food security To strengthen health service delivery	*Nutrition-specific:* Exclusive breastfeeding and complementary feeding Growth monitoring and promotion Nutrition rehabilitation and management of acute malnutrition *Nutrition-sensitive:* Agriculture and food security WASH practices BCC	Raised the proportion of children aged 6–23 months receiving a minimally acceptable diet from 23% in 2011 to 59% in 2015∙ Improved dietary diversity, with the percentage of children aged 6–23 months consuming foods from at least four food groups rising from 47% to 60% in 2015∙ Increased the prevalence of exclusive breastfeeding among children aged < six months from 46% to 69% in 2015∙ Supported Dalit families in target districts, allowing them to earn an additional average of USD 3500 per year (2014–2015) through surplus vegetable and poultry sales
BCC, Behavioral Change Communication; FCHV, Female Community Health Volunteer; IYCF, Infant and Young Child Feeding; MSNP, Multisectoral Nutrition Plan; WASH, Water, Sanitation, and Hygiene.		
(**B**)		
**Programs**	**Key interventions**	**Key outcome measures**
**The National Nutrition Services (established in 2011)** [61,62] To integrate nutrition services into primary healthcare by prioritizing malnutrition prevention and treatment	*Nutrition-specific:* Micronutrient supplementation IYCF Management of severe acute malnutrition *Nutrition-sensitive:* School nutrition programs BCC Training of healthcare providers and community workers to deliver nutrition services	Improved micronutrient status, breastfeeding rates among infants aged 0–6 months Improved nutritional awareness Formulation of national guidelines and policies to support nutrition interventions
**NPAN and NPAN 2 (1997; 2016–2025)** NPAN adopted a multisectoral approach to address malnutrition, involving health, agriculture, education, and social welfare sectors [59] Expanding on NPAN, NPAN2 targets a reduction in stunting from 36% to 25% among children aged < 5 years by 2025 [61]	*Nutrition-specific:* Micronutrient supplementation Therapeutic feeding IYCF practices Food fortification Maternal nutrition support *Nutrition-sensitive:* Food security and agriculture WASH practices Education and awareness campaigns Social safety nets * Policy development	Improvement in the overall nutrition status of its population [62] Notable progress has been made in reducing stunting prevalence, with 2022 data indicating a rate of 26.4% [25] Increased government-led coordination mechanisms at national and sub-national levels [79]
**New School Milk Program (launched in 2023)** To enhance child health while improving school attendance and local dairy sector development [78]	*Nutrition-specific:* Provision of safe and nutritious milk to 60,000 primary school children across 300 schools [78]	While specific data on the New School Milk Programmes’ impact on nutrition and stunting rates in Bangladesh is not yet available, school milk programs in other countries have been shown to support better health and educational outcomes [78]
* Providing food or cash assistance to vulnerable populations to reduce food insecurity. BCC, Behavioral Change Communication; IYCF, Infant and Young Child Feeding; NPAN, National Plan of Action for Nutrition.		
(**C**)		
**Programs**	**Key interventions**	**Key outcome measures**
**National Nutrition Strategy (2011–2020)** [74] To improve nutritional status suitable to each target group, locality, region, and ethnic group	*Nutrition-specific:* Micronutrient supplementation Promotion of exclusive breastfeeding Therapeutic feeding programs *Nutrition-sensitive:* Promotion of food security WASH practices Integration of nutrition into agriculture, education, and social protection policies.	Contributed to reduction in stunting prevalence among children aged < 5 years from 29.3% in 2010 to 19.6% in 2020 Rate of chronic energy deficiency in women of reproductive age declined from 18.5% in 2010 to 9.5% in 2020 Achieved the strategy’s target by reducing the proportion of households with low energy intake (below 1800 kcal) to 5% by 2020
**National School Milk Program** To improve nutrition and enhance the growth of primary school children through daily milk consumption [66]	*Nutrition-specific:* Provision of milk * *Nutrition-sensitive:* Educational campaigns Financial subsidy of milk for low-income families and ethnic minorities	Schoolchildren receiving milk showed a statistically significant greater increase in weight (3.19 vs. 2.95 kg; *p* < 0.001) and height (8.15 vs. 7.88 cm; *p* = 0.008), compared to those not receiving milk [66] Improved children’s nutritional status and increased community awareness of the benefits of regular milk consumption for child development [66]
**Hunger Eradication and Poverty Reduction Program (initiated in 1992)** To reduce hunger and poverty, key contributors to malnutrition and stunting [65] Incorporated economic reforms and investments in child health and family planning [65]	*Nutrition-sensitive:* Substantial allocation of the health budget to nutrition initiatives [65]	Reduction in household poverty rate from 14.2% in 2010 to 8% in 2013 [65] Achieved its Millennium Development Goal of reducing poverty by half, 10 years ahead of the UN’s 2015 target [65]
* Fortifying milk with essential nutrients. UN, United Nations; WASH, Water, Sanitation, and Hygiene.		

## References

[1] Tanner J.M. (1986). Normal growth and techniques of growth assessment. Clin. Endocrinol. Metab..

[2] Jelenkovic A., Sund R., Hur Y.M., Yokoyama Y., Hjelmborg J.V., Moller S., Honda C., Magnusson P.K., Pedersen N.L., Ooki S. (2016). Genetic and environmental influences on height from infancy to early adulthood: An individual-based pooled analysis of 45 twin cohorts. Sci. Rep..

[3] Cohen P., Rogol A.D., Deal C.L., Saenger P., Reiter E.O., Ross J.L., Chernausek S.D., Savage M.O., Wit J.M. (2008). Consensus statement on the diagnosis and treatment of children with idiopathic short stature: A summary of the Growth Hormone Research Society, the Lawson Wilkins Pediatric Endocrine Society, and the European Society for Paediatric Endocrinology Workshop. J. Clin. Endocrinol. Metab..

[4] Grigoletto V., Occhipinti A.A., Pellegrin M.C., Sirchia F., Barbi E., Tornese G. (2021). Definition and prevalence of familial short stature. Ital. J. Pediatr..

[5] Insider B. Average Human Height by Country. https://en.wikipedia.org/wiki/Average_human_height_by_country.

[6] Adair L.S. (2014). Long-term consequences of nutrition and growth in early childhood and possible preventive interventions. Nestle Nutr. Inst. Workshop Ser..

[7] Wallerich L., Fillol A., Rivadeneyra A., Vandentorren S., Wittwer J., Cambon L. (2023). Environment and child well-being: A scoping review of reviews to guide policies. Health Promot. Perspect..

[8] Prendergast A.J., Humphrey J.H. (2014). The stunting syndrome in developing countries. Paediatr. Int. Child. Health.

[9] Robinson P.N., Arteaga-Solis E., Baldock C., Collod-Beroud G., Booms P., De Paepe A., Dietz H.C., Guo G., Handford P.A., Judge D.P. (2006). The molecular genetics of Marfan syndrome and related disorders. J. Med. Genet..

[10] Giustina A., Mazziotti G., Canalis E. (2008). Growth hormone, insulin-like growth factors, and the skeleton. Endocr. Rev..

[11] Mullur R., Liu Y.Y., Brent G.A. (2014). Thyroid hormone regulation of metabolism. Physiol. Rev..

[12] Rogol A.D., Clark P.A., Roemmich J.N. (2000). Growth and pubertal development in children and adolescents: Effects of diet and physical activity. Am. J. Clin. Nutr..

[13] Hvidt J.J., Brix N., Ernst A., Lauridsen L.L.B., Ramlau-Hansen C.H. (2019). Size at birth, infant growth, and age at pubertal development in boys and girls. Clin. Epidemiol..

[14] Gohlke B.C., Bettendorf M., Binder G., Hauffa B., Reinehr T., Dorr H.G., Wolfle J. (2022). Effect of Psychosocial Factors on Growth. Klin. Padiatr..

[15] Daelmans B., Manji S.A., Raina N. (2021). Nurturing Care for Early Childhood Development: Global Perspective and Guidance. Indian Pediatr..

[16] Gao Z., Chen S., Sun H., Wen X., Xiang P. (2018). Physical activity in children’s health and cognition. BioMed Res. Int..

[17] Alves J.G.B., Alves G.V. (2019). Effects of physical activity on children’s growth. J. Pediatr..

[18] Zaffanello M., Pietrobelli A., Cavarzere P., Guzzo A., Antoniazzi F. (2023). Complex relationship between growth hormone and sleep in children: Insights, discrepancies, and implications. Front. Endocrinol..

[19] Sinha P., Guerrant R.L. (2024). The Costly Vicious Cycle of Infections and Malnutrition. J. Infect. Dis..

[20] Black R.E., Victora C.G., Walker S.P., Bhutta Z.A., Christian P., de Onis M., Ezzati M., Grantham-McGregor S., Katz J., Martorell R. (2013). Maternal and child undernutrition and overweight in low-income and middle-income countries. Lancet.

[21] Womack S.R., Beam C.R., Giangrande E.J., Scharf R.J., Tong X., Ponnapalli M., Davis D.W., Turkheimer E. (2023). Nonlinear Catch-Up Growth in Height, Weight, and Head Circumference from Birth to Adolescence: A Longitudinal Twin Study. Behav. Genet..

[22] de Onis M., Branca F. (2016). Childhood stunting: A global perspective. Matern. Child Nutr..

[23] Van Beekum M., Berger J., Van Geystelen J., Hondru G., Som S.V., Theary C., Laillou A., Poirot E., Bork K.A., Wieringa F.T. (2022). The associations between stunting and wasting at 12 months of age and developmental milestones delays in a cohort of Cambodian children. Sci. Rep..

[24] Crookston B.T., Schott W., Cueto S., Dearden K.A., Engle P., Georgiadis A., Lundeen E.A., Penny M.E., Stein A.D., Behrman J.R. (2013). Postinfancy growth, schooling, and cognitive achievement: Young Lives. Am. J. Clin. Nutr..

[25] World Health Organization Data Prevalence on Stunting in Children Under 5. https://www.who.int/data/gho/data/indicators/indicator-details/GHO/gho-jme-stunting-prevalence.

[26] Liu Q., Long Q., Garner P. (2017). Growth monitoring and promotion (GMP) for children in low and middle income countries. Cochrane Database Syst. Rev..

[27] Kuwabara R., Urakami T. (2018). Importance of growth monitoring by a health checkup in detecting growth disorders in young children. Biomed. J. Sci. Tech. Res..

[28] Haymond M., Kappelgaard A.M., Czernichow P., Biller B.M., Takano K., Kiess W., Global Advisory Panel Meeting on the Effects of Growth Hormone (2013). Early recognition of growth abnormalities permitting early intervention. Acta Paediatr..

[29] UNICEF, WHO (2023). UNICEF-WHO-The World Bank: Joint Child Malnutrition Estimates (JME)—Levels and Trends.

[30] Report G.N. Country Nutrition Profiles Nepal. https://globalnutritionreport.org/resources/nutrition-profiles/.

[31] UNICEF (2020). UNICEF Conceptual Framework on Maternal and Child Nutrition. https://www.unicef.org/media/113291/file/UNICEF%20Conceptual%20Framework.pdf.

[32] Alaoui E.H. (2023). Socio-Economic Inequalities, Gender and Malnutrition in Developing Countries. Ph.D. Thesis.

[33] Mandara F., Festo C., Killel E., Lwambura S., Mrema J., Katunzi F., Martin H.D., Elisaria E. (2024). The relationship between feeding practices and stunting among children under two years in Tanzania mainland: A mixed-method approach. Bull. Natl. Res. Cent..

[34] Raman S., Napier-Raman S., Pinzón-Segura M.C. (2024). Exploring cultural influences in perinatal and early childhood nutrition. Rev. Salud Pública.

[35] Sjarif D.R., Yuliarti K., Iskandar W.J. (2019). Daily consumption of growing-up milk is associated with less stunting among Indonesian toddlers. Med. J. Indones..

[36] Ballard O., Morrow A.L. (2013). Human milk composition: Nutrients and bioactive factors. Pediatr. Clin. N. Am..

[37] UNICEF (2024). Nutrition Strategic Direction 2030—North, Middle and East Africa Region.

[38] Cooke R., Goulet O., Huysentruyt K., Joosten K., Khadilkar A.V., Mao M., Meyer R., Prentice A.M., Singhal A. (2023). Catch-Up Growth in Infants and Young Children With Faltering Growth: Expert Opinion to Guide General Clinicians. J. Pediatr. Gastroenterol. Nutr..

[39] Grantham-McGregor S., Cheung Y.B., Cueto S., Glewwe P., Richter L., Strupp B. (2007). Developmental potential in the first 5 years for children in developing countries. Lancet.

[40] Mertens A., Benjamin-Chung J., Colford Jr J.M., Hubbard A.E., van der Laan M.J., Coyle J., Sofrygin O., Cai W., Jilek W., Rosete S. (2023). Child wasting and concurrent stunting in low-and middle-income countries. Nature.

[41] Thurstans S., Sessions N., Dolan C., Sadler K., Cichon B., Isanaka S., Roberfroid D., Stobaugh H., Webb P., Khara T. (2022). The relationship between wasting and stunting in young children: A systematic review. Matern. Child. Nutr..

[42] UNICEF (2022). Severe Wasting: An Overlooked Child Survival Emergency. UNICEF Child Alert Report. https://www.unicef.org/media/120346/file/Wasting%20child%20alert.pdf.

[43] Golden M.H.N. (1994). Is complete catch-up possible for stunted malnourished children?. Eur. J. Clin. Nutr..

[44] Cheung Y.B. (2006). Growth and cognitive function of Indonesian children: Zero-inflated proportion models. Stat. Med..

[45] Alam M.A., Richard S.A., Fahim S.M., Mahfuz M., Nahar B., Das S., Shrestha B., Koshy B., Mduma E., Seidman J.C. (2020). Impact of early-onset persistent stunting on cognitive development at 5 years of age: Results from a multi-country cohort study. PLoS ONE.

[46] Ekholuenetale M., Barrow A., Ekholuenetale C.E., Tudeme G. (2020). Impact of stunting on early childhood cognitive development in Benin: Evidence from Demographic and Health Survey. Egypt. Pediatr. Assoc. Gaz..

[47] Hoddinott J., Behrman J.R., Maluccio J.A., Melgar P., Quisumbing A.R., Ramirez-Zea M., Stein A.D., Yount K.M., Martorell R. (2013). Adult consequences of growth failure in early childhood123. Am. J. Clin. Nutr..

[48] Akseer N., Tasic H., Onah M.N., Wigle J., Rajakumar R., Sanchez-Hernandez D., Akuoku J., Black R.E., Horta B.L., Nwuneli N. (2022). Economic costs of childhood stunting to the private sector in low-and middle-income countries. EClinicalMedicine.

[49] Vaivada T., Akseer N., Akseer S., Somaskandan A., Stefopulos M., Bhutta Z.A. (2020). Stunting in childhood: An overview of global burden, trends, determinants, and drivers of decline. Am. J. Clin. Nutr..

[50] Report G.N. Country Nutrition Profiles Bangladesh. https://globalnutritionreport.org/resources/nutrition-profiles/asia/southern-asia/bangladesh/.

[51] Report G.N. Country Nutrition Profiles Vietnam. https://globalnutritionreport.org/resources/nutrition-profiles/asia/south-eastern-asia/viet-nam/.

[52] Erlyn P., Hidayat B., Fatoni A., Saksono H. (2021). Nutritional interventions by local governments as an effort to accelerate stunting reduction. J. Bina Praja.

[53] Ruel M.T., Alderman H. (2013). Nutrition-sensitive interventions and programmes: How can they help to accelerate progress in improving maternal and child nutrition?. Lancet.

[54] Budhathoki S.S., Bhandari A., Gurung R., Gurung A., Kc A. (2020). Stunting among under 5-year-olds in Nepal: Trends and risk factors. Matern. Child. Health J..

[55] FAO Multi-Sector Nutrition Plan for Accelerating the Reduction of Maternal and Child Under-Nutrition in Nepal 2013–2017. https://www.fao.org/faolex/results/details/en/c/LEX-FAOC143047/.

[56] Exemplars in Global Health What Nepal Do. https://www.exemplars.health/topics/stunting/nepal/what-did-nepal-do.

[57] Siddiqui M.Z., Illiyan A., Akram V., Nigar K. (2024). Revisiting swimming against tide; inequalities in child malnutrition in Nepal. Discov. Glob. Soc..

[58] Namirembe G., Shrestha R., Mezzano J., Ausman L.M., Davis D., Baral K., Ghosh S., Shively G., Webb P. (2021). Effective nutrition governance is correlated with better nutrition outcomes in Nepal. BMC Pediatr..

[59] Adhikari N., Adhikari M., Shrestha N., Pradhananga P., Poudel B., Dhungel S., Joshi P.C., Ide N., Sharma G.N., Shrestha A. (2023). Nutrition and food security in Nepal: A narrative review of policies. Nutr. Rev..

[60] Choufani J., Jamaluddine Z., Cunningham K. (2020). A multisectoral nutrition program in Nepal improves knowledge of dietary diversity, sick child feeding, and handwashing, but not all practices: A program impact pathways mediation analysis. Curr. Dev. Nutr..

[61] Islam M.R., Rahman M.S., Rahman M.M., Nomura S., De Silva A., Lanerolle P., Jung J., Rahman M.M. (2020). Reducing childhood malnutrition in Bangladesh: The importance of addressing socio-economic inequalities. Public Health Nutr..

[62] Nisbett N., Davis P., Yosef S., Akhtar N. (2017). Bangladesh’s story of change in nutrition: Strong improvements in basic and underlying determinants with an unfinished agenda for direct community level support. Glob. Food Secur..

[63] Shamim A.A., Mistry S.K., Irfan N.M. (2019). A Study to Identify the Research Gaps for Effective Implementation of the Second National Plan of Action for Nutrition (NPAN2) in Bangladesh.

[64] Reliefweb JANO’s Nutrition Governance Approach—Multi-Sectoral, Multi-Level and Multi-Stakeholder. https://reliefweb.int/report/bangladesh/janos-nutrition-governance-approach-multi-sectoral-multi-level-and-multi-stakeholder.

[65] Anh N.T.N. (2015). Hunger Eradication and Poverty Reduction in Vietnam: Achievements and Challenges. https://www.freiheit.org/vietnam/report-vietnam-hunger-eradication-and-poverty-reduction-vietnam-achievements-and-challenges.

[66] Hall A., Hanh T.T.M., Farley K., Quynh T.P.N., Valdivia F. (2007). An evaluation of the impact of a school nutrition programme in Vietnam. Public Health Nutr..

[67] Phu P.V., Hoan N.V., Salvignol B., Treche S., Wieringa F.T., Dijkhuizen M.A., Khan N.C., Tuong P.D., Schwartz H., Berger J. (2012). A six-month intervention with two different types of micronutrient-fortified complementary foods had distinct short-and long-term effects on linear and ponderal growth of Vietnamese infants. J. Nutr..

[68] Rocha C., Yeudall F., Moraes A., Yuan Y., Tenkate T., Mendonça M., Duong V.D., Do Huy N., Huyn P., Bao Hoa D.T. (2018). Scaling Up Small-Scale Food Processing for Therapeutic and Complementary Foods for Children in Vietnam.

[69] Joshi G., Chitekwe S. (2019). A Road Map to Nepal’s Multi Sector Nutrition Plan (MSNP) II 2018–2022. https://scalingupnutrition.org/sites/default/files/2021-12/A-Road-Map-to-MSNP-II-in-Nepal.pdf.

[70] Joshi A., Marasini S., Sharma S., Paneru B., Kunwar S., Shrestha A., Shrestha A., Karmacharya B.M. (2024). Scope of work and contributions of female community health volunteers in Nepal’s healthcare sector: A qualitative study. BMJ Open.

[71] Ruducha J., Bhatia A., Mann C., Torlesse H. (2022). Multisectoral nutrition planning in Nepal: Evidence from an organizational network analysis. Matern. Child. Nutr..

[72] Cunningham K., Singh A., Pandey Rana P., Brye L., Alayon S., Lapping K., Gautam B., Underwood C., Klemm R.D. (2017). Suaahara in Nepal: An at-scale, multi-sectoral nutrition program influences knowledge and practices while enhancing equity. Matern. Child. Nutr..

[73] UNICEF Bangladesh: Nutrition for Growth (N4G) Commitment. https://www.unicef.org/bangladesh/en/press-releases/government-bangladesh-commits-improving-nutritional-well-being-mothers-and.

[74] Vietnam National Nutrition Strategy 2021–2030 with Vision to 2040. https://www.n4d.group/wp-content/uploads/2023/02/Vietnam-National-Nutrition-Strategy.pdf.

[75] Khatri R.B., Mishra S.R., Khanal V. (2017). Female community health volunteers in community-based health programs of Nepal: Future perspective. Front. Public Health.

[76] Jomaa L.H., McDonnell E., Probart C. (2011). School feeding programs in developing countries: Impacts on children’s health and educational outcomes. Nutr. Rev..

[77] Berger J., Wieringa F.T., Laillou A., Pham Van P., Dijkhuizen M.A. (2013). Strategies to improve micronutrient status of infants and young children with special attention to complementary foods fortified with micronutrients: Perspectives from Vietnam. Handbook of Food Fortification and Health: From Concepts to Public Health Applications.

[78] Hossain M.S. (2021). School Meals and Child Outcomes in Bangladesh. BUFT J. Bus. Econ. (BJBE).

[79] Saha K.K., Billah M., Menon P., El Arifeen S., Mbuya N.V. (2015). Bangladesh National Nutrition Services: Assessment of Implementation Status.

[80] Nurilah E., Futriani E.S. (2023). Effectiveness Of Supplementary Feeding (PMT) Against Height And Weight Gain Of Stunted Toddlers. Int. J. Health Pharm. (IJHP).

[81] Kristjansson E., Francis D.K., Liberato S., Jandu M.B., Welch V., Batal M., Greenhalgh T., Rader T., Noonan E., Shea B. (2015). Food supplementation for improving the physical and psychosocial health of socio-economically disadvantaged children aged three months to five years: A systematic review. Campbell Syst. Rev..

[82] Semba R.D. (2016). The rise and fall of protein malnutrition in global health. Ann. Nutr. Metab..

[83] Kaur A., Choudhary M., Kapoor S. (2023). Milk as a Functional Food for Health. Functional Foods.

[84] Wiley A.S. (2005). Does milk make children grow? Relationships between milk consumption and height in NHANES 1999–2002. Am. J. Hum. Biol. Off. J. Hum. Biol. Assoc..

[85] Duan Y., Pang X., Yang Z., Wang J., Jiang S., Bi Y., Wang S., Zhang H., Lai J. (2020). Association between dairy intake and linear growth in Chinese pre-school children. Nutrients.

[86] O’Dwyer M., Gillam S. (1995). Children discharged following nutritional rehabilitation: A follow-up study. Trop. Dr..

[87] Sternin M., Sternin J., Marsh D. (2013). Scaling up a poverty alleviation and nutrition program in Vietnam. Scaling Up Scaling Down.

[88] Truong D.T.T., Tran T.H.T., Nguyen T.T.T., Tran V.H.T. (2022). Double burden of malnutrition in ethnic minority school-aged children living in mountainous areas of Vietnam and its association with nutritional behavior. Nutr. Res. Pract..

[89] Harris J., Huynh P., Nguyen H.T., Hoang N., Mai L.T., Tuyen L.D., Nguyen P.H. (2021). Nobody left behind? Equity and the drivers of stunting reduction in Vietnamese ethnic minority populations. Food Secur..

[90] Mbuya N.V., Atwood S.J., Huynh P.N. (2019). Persistent Malnutrition in Ethnic Minority Communities of Vietnam: Issues and Options for Policy and Interventions.

[91] Van Minh H., Mai V.Q., Anh T.T., Duyen N.T., Tuyen L.D., Mai T.T., Phuong H.N., Mustafa T.S., Nwaigwe F., Phuong D.H. (2019). The cost of implementing Vietnam’s national plan of action for nutrition for 2017–2020. AIMS Public Health.

[92] Bhutta Z.A., Akseer N., Keats E.C., Vaivada T., Baker S., Horton S.E., Katz J., Menon P., Piwoz E., Shekar M. (2020). How countries can reduce child stunting at scale: Lessons from exemplar countries. Am. J. Clin. Nutr..

[93] DeTora L.M., Toroser D., Sykes A., Vanderlinden C., Plunkett F.J., Lane T., Hanekamp E., Dormer L., DiBiasi F., Bridges D. (2022). Good Publication Practice (GPP) Guidelines for Company-Sponsored Biomedical Research: 2022 Update. Ann. Intern. Med..

